# Efficient Inference of Parsimonious Phenomenological Models of Cellular Dynamics Using S-Systems and Alternating Regression

**DOI:** 10.1371/journal.pone.0119821

**Published:** 2015-03-25

**Authors:** Bryan C. Daniels, Ilya Nemenman

**Affiliations:** 1 Center for Complexity and Collective Computation, Wisconsin Institute for Discovery, University of Wisconsin, Madison, WI 53715, USA; 2 Departments of Physics and Biology, Emory University, Atlanta, GA 30322, USA; University of Manchester, UNITED KINGDOM

## Abstract

The nonlinearity of dynamics in systems biology makes it hard to infer them from experimental data. Simple linear models are computationally efficient, but cannot incorporate these important nonlinearities. An adaptive method based on the S-system formalism, which is a sensible representation of nonlinear mass-action kinetics typically found in cellular dynamics, maintains the efficiency of linear regression. We combine this approach with adaptive model selection to obtain efficient and parsimonious representations of cellular dynamics. The approach is tested by inferring the dynamics of yeast glycolysis from simulated data. With little computing time, it produces dynamical models with high predictive power and with structural complexity adapted to the difficulty of the inference problem.

## Introduction

Dynamics of cellular regulation are driven by large and intricate networks of interactions at the molecular scale. Recent years have seen an explosive growth in attempts to automatically infer such dynamics, including their functional form as well as specific parameters, from time series of gene expression, metabolite concentrations, or protein signaling data [1–6]. Searching the combinatorially large space of all possible multivariate dynamical systems requires vast amounts of data (and computational time), and realistic experiments cannot sufficiently constrain many properties of the inferred dynamics [7]. Thus detailed, mechanistic models of cellular processes can overfit and are not predictive. Instead, one often seeks *a priori* information to constrain the search space to simpler *ad hoc* models of the underlying complex processes [2, 4, 8]. This can lead to missing defining features of the underlying dynamics, and hence to poor predictions as well.

A promising approach is to forgo mechanistic accuracy in favor of *phenomenological* or *effective* models of the dynamics [1, 9, 10]. Following the recipe that can be traced to Crutchfield and McNamara [11], one defines a sufficiently general (and, hopefully, complete [12]) set of possible dynamics using pre-defined combinations of basis functions. One then uses statistical model selection techniques to select a model that is “as simple as possible, but not simpler” than needed for predictive modeling of the studied system [13]. Such approaches expand traditional statistical modeling to the realm of nonlinear dynamical systems inference. While their derived models may not have an easy mechanistic interpretation, they are parsimonious and often predict responses to yet-unseen perturbations better than microscopically accurate, and yet underconstrained models.

In particular, we have recently implemented such an approach [10] using S-systems (to be described later in this article) as a basis for representing the dynamics. The representation is nested, so that the dynamics can be ordered by an increasing complexity. It is also complete, so that every sufficiently smooth dynamics can be represented with an arbitrary accuracy using large dynamical S-systems, which grow to include complex nonlinearities and dynamical variables unobserved in experiments. Thus inference of the dynamics with this representation is statistically consistent [12] in the sense that, for a large amount of data, the inferred models are guaranteed to become arbitrarily accurate with high probability. The success of such phenomenological modeling is evident from its ability to use simulated noisy data to infer that equations of motion in celestial mechanics are second order differential equations, and to derive Newton’s law of universal gravitation in the process [10]. Similarly, the approach infers parsimonious, phenomenological, and yet highly accurate models of various cellular processes with many hidden variables using about 500 times fewer data than alternative methods that strive for the mechanistic accuracy [10].

And yet the approach in [10] suffers from a high computational complexity, requiring numerical integration of millions of trial systems of differential equations to generalize well. In specific scenarios, it may be useful to trade some of its generality for a substantial computational speedup.

As has been argued in [2, 5], in biological experiments, it is often possible to measure not just expressions of all relevant molecular species, but also their rates of change. In experiments that make use of chemostats [14], for instance, the rates of change are often the quantities of interest, and are measured in many cases more precisely than the absolute concentrations. In such cases, there is the possibility of a large gain of computational speed in model inference because estimates of rates of change can be tested directly, without the requirement of numerical integration of ODEs.

In this article, we show that, in such a scenario, the alternating regression method for inference of S-systems [2], merged with the Bayesian model selection for dynamics [10], infers dynamical models with little computational effort. Importantly, these phenomenological models are parsimonious, nonlinear, and hence predictive even when the microscopically accurate structure of the dynamics is unknown.

In the remainder of the article, we first formalize the problem we study, then introduce the reader to S-systems formalism. We then explain the alternating regression method for inference of the S-systems parameters from data, followed by our implementation of S-systems model selection using Bayesian approaches. Finally, we introduce the biological system on which our approach is tested [15], and present the results of the automated dynamical systems inference in this context.

## Methods

### Problem setup: Inferring biochemical dynamics from data

In a general scenario [10], one needs to infer the deterministic dynamics from a time series of *J*-dimensional vectors of molecular expressions, {*x*_*μ*_}_*i*_ = **x**_*i*_, *μ* = 1…*J*, measured with noise at different time points *t*_*i*_, *i* = 1…*N*. The system may be arbitrarily nonlinear and the measured data vectors **x** may represent only a small fraction of the total number of densely interacting dynamical variables. Intrinsic noise in the system, while possibly important [16], is neglected for simplicity.

We focus on a simplified case [2, 5] in which the measured dynamical variables completely define the system, and where their rates of change, $d{\text{x}}_{i}/dt={\text{x}}_{i}^{\prime }$, and the expected experimental variances of the rates, ${{\text{σ}}^{\prime }}_{i}^{2}$, are also given. Since typical experiments measure variables with higher accuracy than their rates of change, for simplicity, we assume no errors in **x**_*i*_. (This framework can also be straightforwardly extended to include known exogenous variables; for simplicity we do not include this in the example here.) Since the mechanistically accurate form of **X** ^′^ is unknown, we are required then to *approximate* the rate function **X** ^′^:

$$\begin{array}{ccc} \frac{dx}{dt} & = & X^{'}\left(x\right). \end{array}$$ (1)

Knowing ${\text{x}}_{i}^{\prime }$, we can fit the functions **X** ^′^ by minimizing

$$\begin{array}{c} \chi^{2}=\sum_{i=1}^{N}\sum_{\mu =1}^{J}{\left(\frac{x_{i,\mu }^{'}-X_{\mu }^{'}\left(x_{i}\right)}{\sigma_{i,\mu }^{'}}\right)}^{2}, \end{array}$$ (2)

subject to a traditional penalty for the model complexity. Note that here we use the measured values **x**_*i*_ instead of the integrated model values $\tilde{\text{x}}(t_{i})$ as the argument of **X** ^′^.

Different approximation strategies correspond to different functional forms of **X** ^′^. In this paper, we focus on the linear approximation,

$$\begin{array}{c} \frac{dx_{\mu }}{dt}=\sum_{\nu =1}^{J}A_{\mu \nu }x_{\nu }, \end{array}$$ (3)

as well as on S-systems (see below), which can be viewed as linear models in logarithmic space. However, compared to the previous work [2], the analyzed dynamics is not expected to be fitted exactly by either of the model families, resulting in approximate, effective, or phenomenological models. Further, even for a modestly large number of variables, the number of interaction parameters, such as *A*_*μν*_, can be quite large. Thus we additionally study the effects of constraining the interaction model using Bayesian model selection.

Our **goals** are to understand (1) the accuracy afforded by the nonlinear S-system approach for phenomenological modeling, especially compared to a simpler linear regression, (2) the computational efficiency of the S-systems alternating regression method compared to more complex fitting approaches in [5, 10], and (3) whether selection of parsimonious models using the Bayesian approach, first tried for dynamical systems in Ref. [10], similarly provides an improvement over fitting a complex, fully connected interaction model.

### S-systems formalism

The textbook representation of biochemical dynamics uses ordinary differential equations in the *mass-action* form, where the rate laws are differences of production (*G*) and degradation (*H*) terms,

$$\begin{array}{c} \frac{dx_{\mu }}{dt}=G_{\mu }\left(x\right)-H_{\mu }\left(x\right). \end{array}$$ (4)

In their turn, *G* and *H* are products of integer powers of expressions of other chemical species, where the powers represent stoichiometry. For example, if a certain chemical *μ* is produced by a bimolecular reaction involving one molecule of *ν* and two molecules of *λ*, then its mass-action production term is $G_{\mu }=\alpha_{\mu }x_{\nu }x_{\lambda }^{2}$, where *α*_*μ*_ is the reaction rate.

In what became known as the S-systems formalism, Savageau and colleagues generalized this form to non-integer powers of expressions [17]. That is,

$$\begin{array}{c} G_{\mu }\left(x\right)=\alpha_{\mu }\prod_{\nu =1}^{J}x_{\nu }^{g_{\mu \nu }},\;H_{\mu }\left(x\right)=\beta_{\mu }\prod_{\nu =1}^{J}x_{\nu }^{h_{\mu \nu }}. \end{array}$$ (5)

The S-system is a *canonical representation* of nonlinear biochemical dynamics since, in a process called *recasting*, any dynamics, Equation (1), with **X** ^′^ written in terms of elementary functions can be rewritten in this power-law form by introducing auxiliary dynamical variables and initial value constraints in a certain way [17]. Further, since any sufficiently smooth function can be represented as a series of elementary functions (e. g., Taylor series), a recasting into an S-system of a sufficient size can approximate any such deterministic dynamics. While a detailed review of recasting is beyond the scope of this article, here we give a few examples. First, the classic Michaelis-Menten enzymatic kinetics, $x_{1}^{\prime }=Ax_{1}/(B+x_{1})$, can be recast by introducing one hidden variable *x*_2_ as

$$\begin{array}{cc} x_{1}^{'} & =Ax_{1}x_{2}^{-1},\;x_{2}=B+x_{1}. \end{array}$$ (6)

Similarly, the dynamics $x_{1}^{\prime }=\sin x_{1}$ has a representation

$$\begin{array}{c} x_{1}^{'}=x_{2},\;x_{2}^{'}=x_{3}x_{2},\;x_{3}^{'}=-x_{2}^{2}, \end{array}$$ (7)

where *x*_2_ = sin*x*_1_, and *x*_3_ = cos*x*_1_. (This can be checked by observing that $x_{2}^{\prime }=x_{1}^{\prime }\cos x_{1}$ and $x_{3}^{\prime }=-x_{1}^{\prime }\sin x_{1}$.) Note that, since the exponents are not constrained to be positive or integer, dynamics in this class are generally ill-defined when variables are not positive.

The power-law structure of *G* and *H* takes a particularly simple form if viewed in the space of logarithms of the dynamical variables, *ξ*_*ν*_ = log*x*_*ν*_:

$$\begin{array}{c} \log G_{\mu }\left(x\right)=\log\alpha_{\mu }+\sum_{\nu =1}^{J}g_{\mu \nu }\xi_{\nu },\;\;\log H_{\mu }\left(x\right)=\log\beta_{\mu }+\sum_{\nu =1}^{J}h_{\mu \nu }\xi_{\nu }. \end{array}$$ (8)

Thus S-systems can be rationalized even when the set of the modeled variables is fixed, not allowed to be enlarged, and hence recasting is impossible: S-systems are the first order expansion of the logarithms of the production/degradation terms in the logarithms of the concentration variables. Arbitrary dynamics can be approximated again by adding a sufficient number of higher order terms in this logarithmic Taylor expansion, just like they can be added to Equation (3). However, it has been argued that typical biochemical kinetics laws remain linear for broader ranges in the logarithmic, rather than the linear space [18]. At the same time, they can produce richer dynamics than that of simple linear models. Thus S-systems have a potential to be more effective in approximating biological dynamics. Verifying this assertion is one of the goals of this article.

### Alternating regression for S-systems inference

The key observation that leads to efficient inference of S-systems is that each of the production and degradation terms is linear in its parameters in log-space, Equation (8) [2]. Specifically, if we know the current concentrations **x** and their time derivatives **x** ^′^, and hold constant the parameters in one of the two terms (say, *G*), the parameters in the other term (*H*) can be fit using linear regression. The alternating regression approach, first implemented in [2] for models that can be fitted exactly by S-systems, simply iteratively switches between linearly fitting parameters in the two terms. Thus, for each S-system model, defined by which interactions *g*_*μν*_ and *h*_*μν*_ are allowed to be nonzero, the inference consists of the following two steps repeated until convergence:

1. *Fit production terms, holding degradation fixed*. That is, in Equation (4), solve for *α*_*μ*_ and *g*_*μν*_ using a linear regression (see below), while holding *β*_*μ*_ and *h*_*μν*_ fixed.
2. *Fit degradation terms, holding production fixed*. Same, but swapping (*α*_*μ*_,*g*_*μν*_) and (*β*_*μ*_,*h*_*μν*_).

(Note that, due to the nonlinear nature of the transformation to log-space, we are not guaranteed to have a smaller *χ* ^2^ in linear space after the linear regression in log-space. In practice, we find reliable convergence given a modest quantity of data.)

To implement this logarithmic linear regression, we define

$$\begin{array}{c} Y^{\left(G\right)}=\log\left(H+x^{'}\right),\;Y^{\left(H\right)}=\log\left(G-x^{'}\right), \end{array}$$ (9)

so that in the regression in the two cases we are attempting, we are looking for parameters *α*,*β*,*g*, and *h* that satisfy, for every measured timepoint *t*_*i*_,

$$\begin{array}{c} Y_{i,\mu }^{\left(G\right)}=\log\alpha_{\mu }+\sum_{\nu }g_{\mu \nu }\xi_{i,\nu },\;Y_{i,\mu }^{\left(H\right)}=\log\beta_{\mu }+\sum_{\nu }h_{\mu \nu }\xi_{i,\nu }. \end{array}$$ (10)

We define parameter matrices *P* with a row for the prefactors log*α* and log*β* followed by the matrix of the exponent parameters:

$$\begin{array}{cc} P_{\kappa =1,\mu }^{\left(G\right)}=\log\alpha_{\mu }, & P_{\kappa =1+\nu ,\mu }^{\left(G\right)}=g_{\mu \nu }; \end{array}$$ (11)

$$\begin{array}{cc} P_{\kappa =1,\mu }^{\left(H\right)}=\log\beta_{\mu }, & P_{\kappa =1+\nu ,\mu }^{\left(H\right)}=h_{\mu \nu }. \end{array}$$ (12)

Then, for both *H* and *G*, the problem becomes a linear regression in which we want to minimize the following modified *χ* ^2^ with respect to *P*:

$$\begin{array}{c} {\tilde{\chi }}_{Y}^{2}=\sum_{\mu ,i}{\left[W_{i,\mu }\left(Y_{i,\mu }-\sum_{\kappa }D_{i,\kappa }P_{\kappa ,\mu }\right)\right]}^{2}+\sum_{\mu ,\kappa }\frac{P_{\kappa ,\mu }^{2}}{\varsigma_{p}^{2}}, \end{array}$$ (13)

where the last term corresponds to Gaussian priors on each parameter with mean 0 and variance $\varsigma_{p}^{2}$ (in our example below, we set *ς*_*p*_ = 10^−1^). Here the design matrix *D* combines the prefactor parameters and the data,

$$\begin{array}{c} D_{i,\kappa =1}=1,\;D_{i,\kappa =1+\nu }=\xi_{i,\nu }, \end{array}$$ (14)

and the matrix *W* weights each residual according to its uncertainty in log-space,

$$\begin{array}{c} W_{i,\mu }=\frac{1}{\sigma_{i,\mu }^{'}}\frac{d\exp Y_{i,\mu }}{dY_{i,\mu }}=\frac{\exp Y_{i,\mu }}{\sigma_{i,\mu }^{'}}. \end{array}$$ (15)

Finally, elements of *Y* are undefined when the arguments of the logs in Equation (9) become negative, corresponding to derivatives that cannot be fit by modifying only *G* or *H* at one time. In these cases, we zero the corresponding weight *W*_*i*,*μ*_, effectively removing these datapoints from the regression at a particular iteration. This can be a bigger problem, in principle, for approximate approaches than it was for the case when S-systems could fit the data exactly [2]. In practice, the number of such datapoints quickly becomes small or zero after just a few iterations.

The linear regression can be solved separately for each species *μ*. In matrix form, extremizing the *μ*th term of ${\tilde{\chi }}_{Y}^{2}$ in Equation (13) produces the maximum likelihood parameters for species *μ*:

$$\begin{array}{c} P_{\mu }={\left({\overset{ˇ}{D}}_{\mu }^{T}{\overset{ˇ}{D}}_{\mu }+\frac{1}{\varsigma_{p}^{2}}I\right)}^{-1}{\overset{ˇ}{D}}_{\mu }^{T}{\overset{ˇ}{Y}}_{\mu }, \end{array}$$ (16)

where *I* is the identity matrix, and (*Ď*_*μ*_)_*i*,*ν*_ = *W*_*i*,*μ*_ *D*_*i*,*ν*_, (*Y̌*_*μ*_)_*i*_ = *W*_*i*,*μ*_ *Y*_*i*,*μ*_.

To perform the regression with some parameters fixed, it is convenient to let all matrices remain the same shape instead of removing rows and columns corresponding to parameters fixed at the current iteration. To accomplish this, we define the binary matrix *θ* that is the same shape as *P* and contains a 1 when the corresponding parameter is to be optimized and a 0 when it is not. Because in our model the default fixed value for each parameter is 0, we arrive at (*Ď*_*μ*_)_*iν*_ = *W*_*i*,*μ*_ *D*_*i*,*ν*_ *θ*_*μ*,*ν*_ and

$$\begin{array}{c} P_{\mu }={\left({\overset{ˇ}{D}}_{\mu }^{T}{\overset{ˇ}{D}}_{\mu }+\frac{1}{\varsigma_{p}^{2}}I+\left(1-\theta_{\mu }\right)\delta_{\mu ,\nu }\right)}^{-1}{\overset{ˇ}{D}}_{\mu }^{T}{\overset{ˇ}{Y}}_{\mu }, \end{array}$$ (17)

where the *δ*_*μ*,*ν*_ term removes singularities corresponding to the fixed parameters.

In our experiments, the alternating regression typically converged to a relative tolerance of 10^−2^ in a few tens of iterations. This made it not much slower than the simple linear regression (and many orders of magnitude faster than approaches of Refs. [5, 10]), while preserving the ability to fit nonlinearities pervasive in biological systems.

### Adaptive Bayesian dynamical model selection

Adaptive Bayesian model selection defines an *a priori* hierarchy of models with an increasing complexity and selects the one with the largest Bayesian likelihood given the data. This minimizes the generalization error [12, 19, 20]. Generally, one needs to define such a hierarchy over the number of interactions among species, the nonlinearity of the interactions, and the number of unobserved species in the model [10]. Here we have a fixed number of species, and the situation is simpler.

Indeed, different S-systems models can be characterized by “who interacts with whom”—the identity of those exponential parameters *g*_*μν*_ and *h*_*μν*_ that are allowed to be nonzero. Then the complexity hierarchy over S-systems can be defined by gradually allowing more interactions, so that the production and degradation of species depends on more and more of (other) species. Specifically, we start with the simplest model with 2*J* parameters in which only *β*_*μ*_ and *g*_*μμ*_ need to be inferred from data. Next *h*_*μμ*_ are added to the list of inferable variables, and then *α*_*μ*_ for each *μ*. Next are *g*_*μμ*_ connections starting with the nearest neighbors [*μ*−*ν* = 1 (mod *J*)], then the next-nearest neighbors [*μ*−*ν* = 2 (mod *J*)], and so on until all *g*_*μν*_ are included. Finally we add *h*_*μν*_ connections in the same order. The final, largest model contains 2*J*(*J*+1) parameters. While in the above example the order of the variables and hence the hierarchy is pre-defined, a random order is also possible. As has been argued elsewhere [10], a random hierarchy is still better than no hierarchy at all. In the Results section, we test both the nearest neighbor hierarchy and a series of random hierarchies.

With the hierarchy defined, we calculate the posterior log-likelihood 𝓛 of each model *M* within the S-systems family in the usual way [19]. We expand the log-likelihood of the model to the lowest order in 1/*N*, that is, as a quadratic form near the maximum likelihood values determined by alternating regression. This extends the Bayesian Information Criterion (BIC) [21] by explicitly incorporating parameter sensitivities and priors over the parameters, giving

$$\begin{array}{c} ℒ\left(M\right)\equiv -\frac{1}{2}{\tilde{\chi }}^{2}\left(P_{\mathrm{best}}\right)-\frac{1}{2}\sum_{\mu }\log\lambda_{\mu }-\frac{1}{2}\sum_{k}\log\varsigma_{k}^{2}. \end{array}$$ (18)

Here ${\tilde{\chi }}^{2}=\chi^{2}+\sum_{k}P_{k}^{2}/\varsigma_{k}^{2}$ with *χ* ^2^ defined as in Equation (2), *P*_best_ represents the maximum likelihood parameters found in the alternating regression, and $\varsigma_{k}^{2}$ are the *a priori* parameter variances. Finally, *λ*_*μ*_ are the eigenvalues of the Hessian of the posterior log-likelihood around the maximum likelihood value, which we estimate numerically. We then maximize 𝓛(*M*) over all possible models in the S-systems structure to “select” a parsimonious model with a high generalization power.

Note that some eigenvectors have $\lambda_{\mu }\sim 1/\varsigma_{p}^{2}=o(1)$. These directions in parameter space are *sloppy*[7], in the sense that they cannot be inferred from the data because they do not affect the probability of the data. Thus we define the number of *effective*, or *relevant* parameters in the model relative to the data as the number of eigenvectors with *λ*_*μ*_ > 1. It will be instructive to see how this number for the “selected” model changes as a function of various properties of the training data.

A software implementation of the adaptive model selection algorithm can be found under the project “Sir Isaac” on github: http://github.com/EmoryUniversityTheoreticalBiophysics/SirIsaac.

## Results

**Fig 1:**
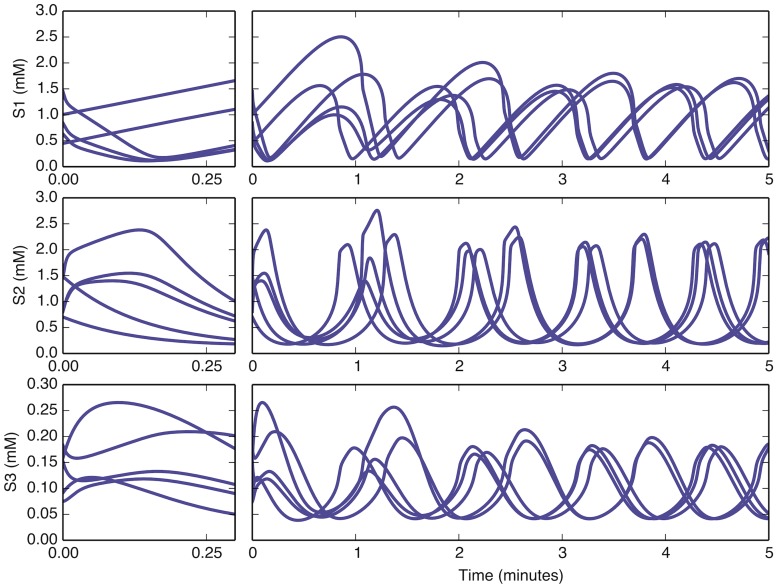
Representative trajectories for the first three species in the yeast glycolysis model. Trajectories are shown for five initial conditions randomly chosen from the ranges in Table 2. The system’s behavior is characterized by a stable limit cycle with a period of about one minute, with transients that subside on the timescale of seconds to minutes. At left are zoomed portions of the trajectory showing fast transients. The period of the oscillation is independent of the initial condition, while the phase depends on it.

The model of oscillations in yeast glycolysis [15] has become a standard test case for biochemical dynamics inference [3, 10]. We use it here as well to analyze the performance of our adaptive S-systems approach. The biochemical details are not critical to our current purpose; we instead take the model as an example of complicated nonlinear dynamics typical of biological systems. The model consists of ODEs for the concentrations of 7 biochemical species:

$$\begin{array}{ccc} \frac{dS_{1}}{dt} & = & J_{0}-\frac{k_{1}S_{1}S_{6}}{1+{\left(S_{6}/K_{1}\right)}^{q}},\\ \frac{dS_{2}}{dt} & = & 2\frac{k_{1}S_{1}S_{6}}{1+{\left(S_{6}/K_{1}\right)}^{q}}-k_{2}S_{2}\left(N-S_{5}\right)-k_{6}S_{2}S_{5},\\ \frac{dS_{3}}{dt} & = & k_{2}S_{2}\left(N-S_{5}\right)-k_{3}S_{3}\left(A-S_{6}\right),\\ \frac{dS_{4}}{dt} & = & k_{3}S_{3}\left(A-S_{6}\right)-k_{4}S_{4}S_{5}-\kappa \left(S_{4}-S_{7}\right),\\ \frac{dS_{5}}{dt} & = & k_{2}S_{2}\left(N-S_{5}\right)-k_{4}S_{4}S_{5}-k_{6}S_{2}S_{5},\\ \frac{dS_{6}}{dt} & = & -2\frac{k_{1}S_{1}S_{6}}{1+{\left(S_{6}/K_{1}\right)}^{q}}+2k_{3}S_{3}\left(A-S_{6}\right)-k_{5}S_{6},\\ \frac{dS_{7}}{dt} & = & \psi \kappa \left(S_{4}-S_{7}\right)-kS_{7}, \end{array}$$ (19)

where the parameters for the model are taken from [15] and listed in Table 1. Representative trajectories from this dynamical system are displayed in Fig. 1. Notice that, for the first few minutes, the system explores a large volume in the phase space, and it settles down onto a much simpler limit-cycle behavior a few minutes into the process.

**Table 1:** Yeast glycolysis model parameters.

*J*_0_	2.5	mM min^−1^	*k*	1.8	min^−1^
*k*_1_	100.	mM^−1^ min^−1^	*κ*	13.	min^−1^
*k*_2_	6.	mM^−1^ min^−1^	*q*	4	
*k*_3_	16.	mM^−1^ min^−1^	*K*_1_	0.52	mM
*k*_4_	100.	mM^−1^ min^−1^	*ψ*	0.1	
*k*_5_	1.28	min^−1^	*N*	1.	mM
*k*_6_	12.	mM^−1^ min^−1^	*A*	4.	mM

Parameters for the yeast glycolysis model defined in Equations (19), from [15].

We evaluate the performance of three types of expansions of **X** ^′^ for the yeast oscillator: a linear representation, Equation 3, a fully-connected S-system with all 2*J*(*J*+1) = 112 parameters fit, and an adaptive S-system with a number of fit parameters that depends on the given data. To create training data, we choose *N* random initial conditions uniformly from ranges shown in Table 2 [5], integrate each forward in time using Equations (19) until time *t*_*i*_ chosen uniformly from time 0 to time *T*, and use the values of **x** and **x** ^′^ at *t*_*i*_ as the input data. To simulate experimental noise, we corrupt each evaluated rate by a rather large Gaussian noise with the standard deviation of 0.5*σ*_*x*^′^_*μ*__, where *σ*_*x*^′^_*μ*__ is the standard deviation of the rate $x_{\mu }^{\prime }$ sampled over long-time behavior of each species, also shown in Table 2. Finally, to evaluate performance in predicting derivatives, we create out-of-sample test data using the same method, with timepoints ranging from *t* = 0 to 5 minutes. For testing, we force all inference methods to extrapolate rather than interpolate by choosing a wider range of initial conditions, as described in Table 2. In this setup, the difficulty of the inference problem is adjusted by varying *T*. Indeed, for *T* = 5, much of the training data is close to the low-dimensional attractor. Since the test data is very similar, the inference only needs to learn the structure of the derivatives near the attractor in this case. In contrast, small *T* forces the algorithms to approximate the system over a larger volume of the phase space, which is harder since nonlinearities start playing a stronger role.

**Table 2:** Specification of training data.

**SPECIES**	**IC RANGE** (mM)	***σ*_*x*^′^_** (mM/min)
*S*_1_	[0.15, 1.60]	1.8442
*S*_2_	[0.19, 2.16]	3.0449
*S*_3_	[0.04, 0.20]	0.1438
*S*_4_	[0.10, 0.35]	0.2746
*S*_5_	[0.08, 0.30]	0.1153
*S*_6_	[0.14, 2.67]	3.4437
*S*_7_	[0.05, 0.10]	0.0489

Ranges of initial conditions (matching [5]) and the experimental noise (chosen as 1/2 the standard deviation of derivatives sampled from the long-time behavior of each species) for training data. In test data, the high ends of initial condition ranges are changed to increase the size of each range by 25%, forcing the fits to extrapolate, and not just to interpolate.

**Fig 2:**
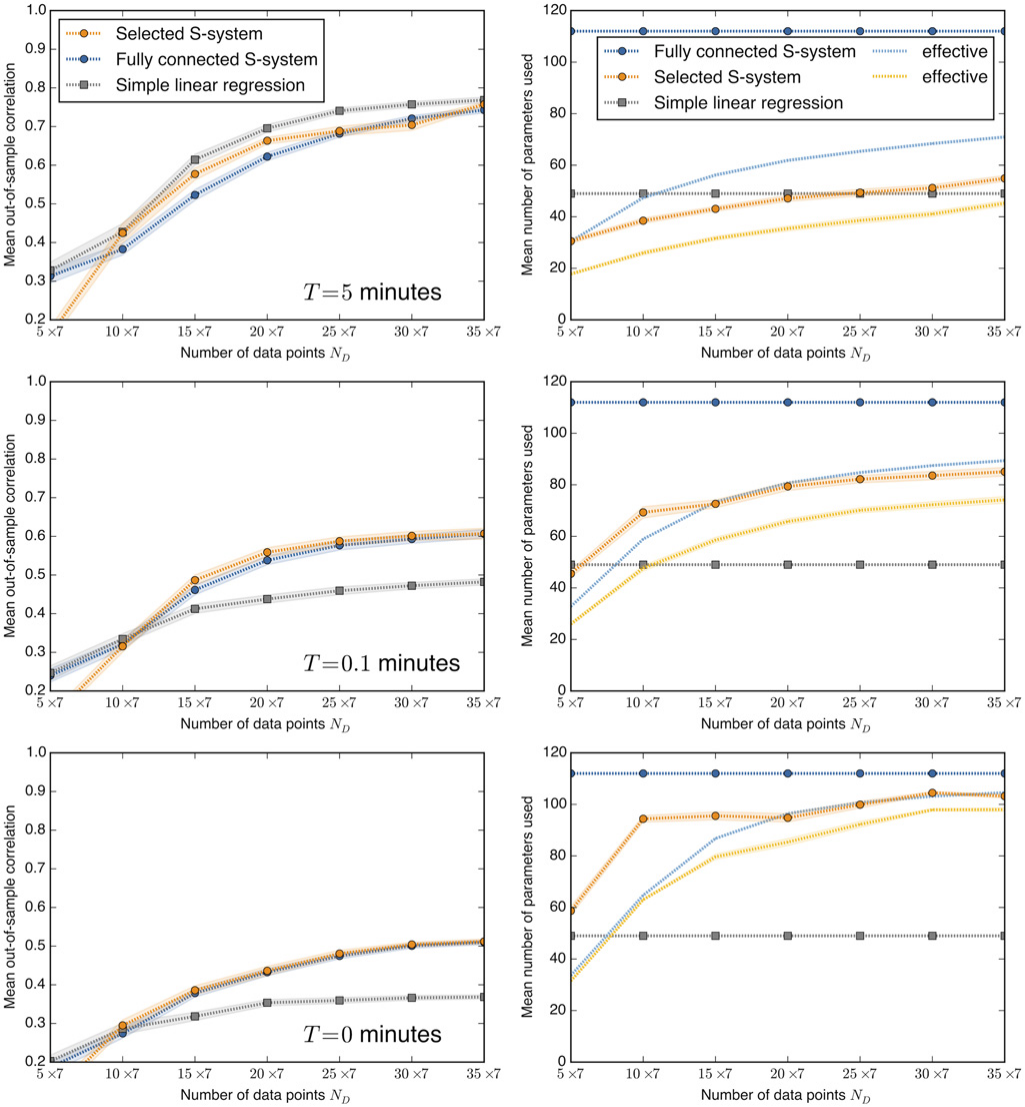
Inference results for yeast oscillator model. (Left column) Mean correlation of predicted and true derivative values on out-of-sample test data versus the amount of data, *N* (since there are 7 species, the total number of data points is 7×*N*), for each of three inference methods. The mean is taken over the 7 species and 100 different realizations of the training data. Shaded areas represent the standard deviation of the mean over the realizations. (Right column) The nominal and the effective (that is, stiff, or determined by the data) number of parameters used in each fit. The fully connected S-system and simple linear regression each have a constant nominal number of parameters, while the nominal number of parameters in the selected S-system adapts to the data. The effective number of parameters is always data dependent. Rows correspond to values of *T*, the upper limit on the range of time values used in training (test data always use *T* = 5 minutes). As described in the text, the complexity of the inference task is higher for lower *T*. In each case, the adaptive S-system model performs at least as well as the other approaches. Nonlinearity of S-systems makes them usable even for the complex inference task, where the range of variables is large and the nonlinear effects are important, so that the linear regression model fails (*T* = 0 min).

The top left plot in Fig. 2 displays the performance of each method in the easiest case (with *T* = 5 minutes), as measured by the mean correlation between predicted and actual out-of-sample derivatives, and as a function of the number of data points *N* in the training set. In this case, much of the training/testing dynamics falls onto the simple, low-dimensional attractor (cf. Fig. 1), and the resulting oscillations are well-fit by the linear regression, though the adaptive S-system method performs nearly as well. On the contrary, since the simple dynamics near the attractor does not require a complex model, the fully connected S-system overfits and performs significantly worse. Indeed, the linear regression and the adapted S-system make their predictions with far fewer parameters than the fully connected model (cf. top right panel in Fig. 2). For comparison, notice also that the approaches requiring identification of a true microscopic model using variable and rate time series [5] or building a phenomenological model using only variable time series [10] both required orders of magnitude more data and computing time to achieve the same level of out-of-sample correlation of 0.6 to 0.8.

Increasing the difficulty of the inference problem by decreasing *T* forces approximating the system over a broader, more nonlinear range. This more severely degrades the predictions of the simple linear regression than those of the S-systems, as displayed in the bottom two rows of Fig. 2. With *T* = 0.1 minutes, the selected S-system is the best performer, slightly better than the fully connected S-system, and with significantly fewer nominal and effective parameters. With *T* = 0, the number of parameters used in the selected S-system approaches the full model. Both are equivalent and perform better than the simple linear regression, as we expect if they are better able to fit the nonlinearities present in the original model.

**Fig 3:**
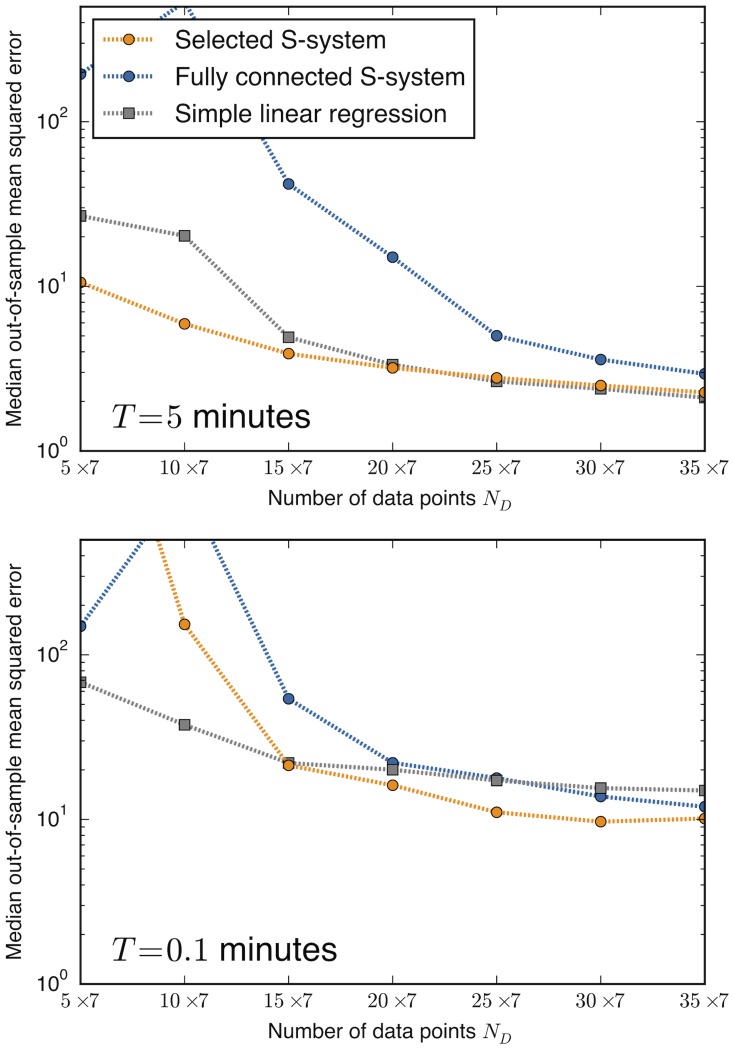
Mean-squared error demonstrates overfitting of fully-connected model. Plotted are the median mean-squared errors measured using each inference method for the cases of *T* = 5 and 0.1 minutes (corresponding to the top two plots in Fig. 2). Decreased performance due to overfitting is evident for the fully-connected S-system models (blue). Squared errors are standardized for each species by dividing by the square of the standard deviations listed in Table 2.

A different measure of performance, the mean-squared error of predicted rates, reaffirms these conclusions. As shown in Fig. 3, the median mean-squared error also more clearly demonstrates the effect of overfitting, with severely degraded performance for the fully-connected S-system at small *N*.

**Fig 4:**
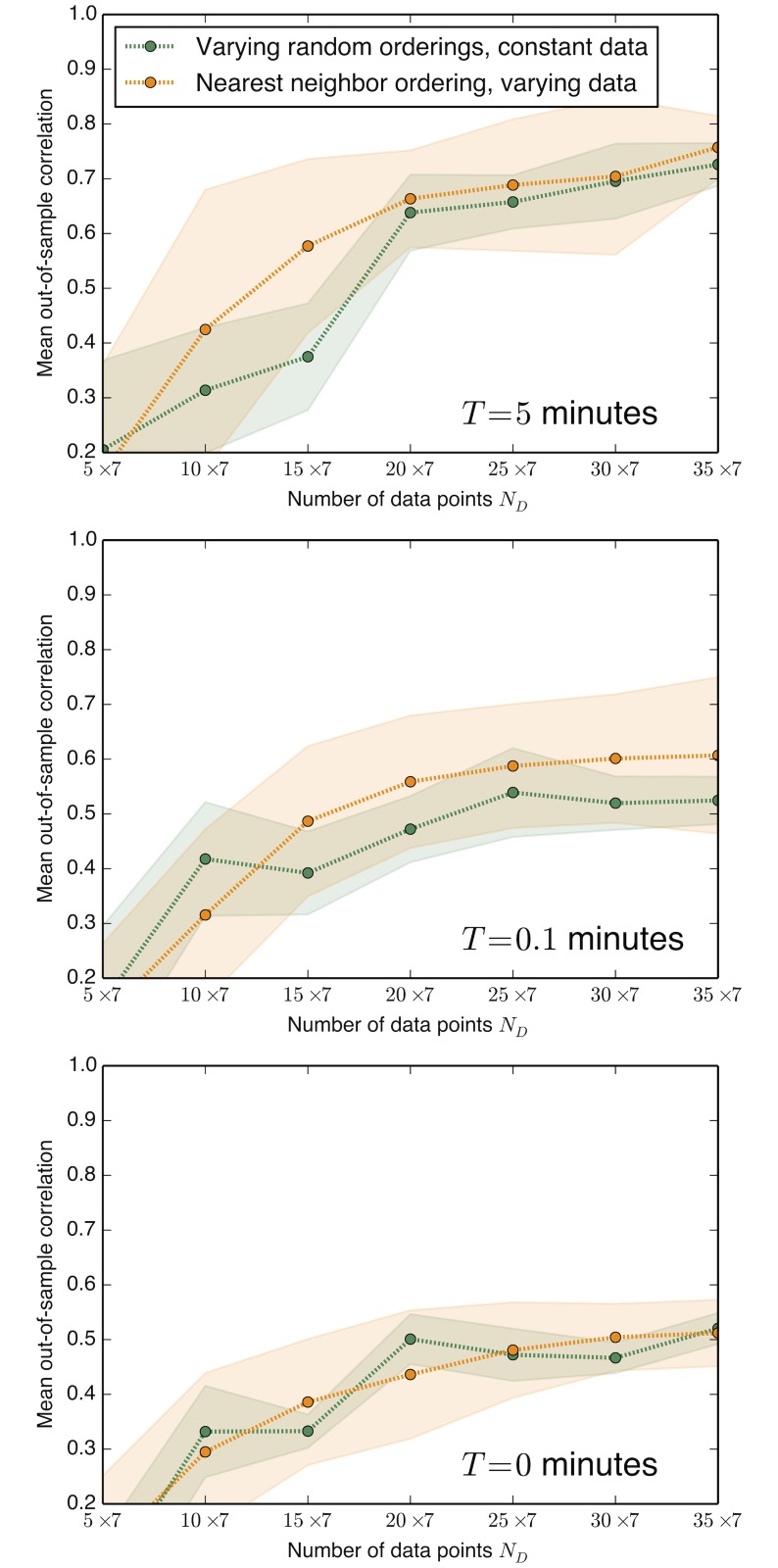
Testing different model hierarchies using the yeast oscillator model. As in Fig. 2, here we plot the mean correlations of predicted and true derivative values on out-of-sample test data versus the amount of data, *N*. We compare different orderings of adding parameters in the model hierarchy. The variance in performance over different orderings of parameters in a random hierarchy (standard deviations plotted in green) is generally smaller than the variance caused by different random realizations of the training data for the single nearest neighbor ordering used in Fig. 2 (standard deviations plotted in orange).

Finally, we test the effects of changing the choice of the model hierarchy, corresponding to changing the order of adding parameters to the model. Fig. 4 demonstrates that the variance due to the choice of the hierarchy is generally smaller than the variance due to the randomness in the sampling of the training data. Thus while restricting the model search to *some* single hierarchy is important, the *specific* choice of hierarchy is not crucial.

## Discussion

In this work we have proposed a new and fast method for inference of the underlying biochemical dynamics from experiments that measure concentrations and rates of changes of all relevant molecular species, such as one would obtain from, for example, cultures in a chemostat. In a previous paper [10], we addressed the case that does not assume measurements of derivatives, nor measurements of all important dynamic variables. Here, we focus on a simpler yet relevant case with stricter assumptions, one that allows for much more efficient inference.

The approach is based on the S-systems formalism, and uses alternating regression and Bayesian model selection for efficient and parsimonious inference. Our results confirm that the alternating regression approach for inferring phenomenological, approximate dynamics using S-systems retains the computational efficiency of simple linear regression, while including nonlinearities that allow it to make better predictions in a typical test case from systems biology. In addition, we generalize the approach of [2] to include adaptive dynamical model selection [10], which allows inference of parsimonious S-systems with an optimal complexity. When the dynamics are more simple (as in the cases with *T* > 0 in Fig. 2), this allows the S-system to use far fewer parameters and avoid overfitting. When dynamics become more nonlinear (as in the case with *T* = 0 in Fig. 2), the approach makes use of more parameters to obtain better predictions than a simple linear model.

We note that our model selection criterion in Equation (18) resembles BIC, but is more general, equivalent to a Gaussian estimate of the full posterior capturing the two lowest order corrections in a large *N* limit. Traditional BIC assumes each parameter contributes equally to the Bayes factor, which is roughly valid in simpler models. This fails spectacularly in complex nonlinear ODE models, with typically widely varying sensitivities in different parameter space directions [7]. This crucial generalization allows such a semi-analytic approach to be useful for a broad class of problems, without reliance on MCMC sampling methods that are much slower.

Inferring the true microscopic structure of the yeast glycolysis dynamics is possible, but requires *N* ~ 10^4^ measurements [5]. Here we demonstrated that, when the structure is unknown *a priori* and cannot be inferred given the limited data set size, *N* ~ 10^1^, a phenomenological expansion of the dynamics can still be useful for making predictions about the system’s behavior. We have described one such representation based on S-systems that is extremely computationally efficient, more predictive than linear models through its incorporation of realistic nonlinearities, and parsimonious through its use of adaptive model selection.
